# Relationship of Serum Trimethylamine N-Oxide (TMAO) Levels with early Atherosclerosis in Humans

**DOI:** 10.1038/srep26745

**Published:** 2016-05-27

**Authors:** Elko Randrianarisoa, Angela Lehn-Stefan, Xiaolin Wang, Miriam Hoene, Andreas Peter, Silke S Heinzmann, Xinjie Zhao, Ingmar Königsrainer, Alfred Königsrainer, Bernd Balletshofer, Jürgen Machann, Fritz Schick, Andreas Fritsche, Hans-Ulrich Häring, Guowang Xu, Rainer Lehmann, Norbert Stefan

**Affiliations:** 1 Department of Internal Medicine IV, University of Tübingen, Germany; 2 Institute of Diabetes Research and Metabolic Diseases (IDM) of the Helmholtz Center Munich at the University of Tübingen, Tübingen, Germany; 3 German Center for Diabetes Research (DZD), 85764 München-Neuherberg, Germany; 4 CAS Key Laboratory of Separation Science for Analytical Chemistry, Dalian Institute of Chemical Physics, Chinese Academy of Sciences, Dalian, China; 5 Research Unit Analytical BioGeoChemistry, Helmholtz Zentrum München, 85764 Neuherberg, Germany; 6 Department of General, Visceral and Transplant Surgery, University of Tübingen, Germany; 7 Section on Experimental Radiology, University of Tübingen, Germany

## Abstract

Circulating trimethylamine N-Oxide (TMAO) levels predict cardiovascular disease (CVD), possibly by impacting on cholesterol metabolism and oxidative stress. Because hepatic TMAO production is regulated by insulin signalling and it is unclear whether and to what extent circulating TMAO levels associate with CVD risk, independently of insulin resistance and its important determinants fatty liver and visceral obesity, we have now addressed this question in 220 subjects who participated in the Tübingen Lifestyle Intervention Program. Visceral fat mass (r = 0.40, p < 0.0001), liver fat content (r = 0.23, p = 0.0005) and TMAO levels (r = 0.26, p < 0.0001) associated positively, and insulin sensitivity associated negatively (r = −0.18, p = 0.009) with carotid intima-media thickness (cIMT). Higher TMAO levels (std.−Beta 0.11, p = 0.03) predicted increased cIMT, independently of age, sex and visceral fat mass. While during the lifestyle intervention most cardiovascular risk parameters improved, mean TMAO levels did not change (p = 0.18). However, cIMT decreased significantly (p = 0.0056) only in subjects in the tertile with the largest decrease of TMAO levels (>20%). We provide novel information that increased serum TMAO levels associate with increased cIMT, independently of established cardiovascular risk markers, including insulin resistance, visceral obesity and fatty liver. Furthermore, the decrease of cIMT during a lifestyle intervention may be related to the decrease of TMAO levels.

The identification of a meta-organismal pathway involving dietary intake, gut microbiota and liver metabolism has raised much interest in the cardiometabolic field of research^1^. The group of Stanley Hazen provided strong support that metabolism of dietary phosphatidylcholine and L-carnitine by intestinal microbiota, resulting in the formation of the metabolite trimethylamine and its hepatic conversion to trimethylamine N-oxide (TMAO), induces atherosclerosis and that high TMAO levels predict an increased risk of cardiovascular disease (CVD)^2^,^3^,^4^,^5^. Another group also found elevated TMAO concentrations to independently predict coronary atherosclerosis and mortality in patients with chronic kidney disease^6^. However, although TMAO levels were found to be increased in hyperglycemic subjects and patients with impaired kidney function in other studies, they were not associated with all-cause mortality, cardiovascular death, or hospitalizations^7^ or history of myocardial infarction, the angiographically assessed presence of coronary heart disease or incident cardiovascular events during 8 years of follow-up^8^.

Importantly, Hazen’s group and others found the flavin mono-oxygenase 3 (FMO3) to play a crucial role in the regulation of circulating blood TMAO levels and in the promotion of atherosclerosis and cardiovascular disease in mice^9^,^10^,^11^. Interestingly, very recent studies suggest that FMO3 may also directly, and independently of TMAO levels, induce hyperglycemia, hyperlipidemia and atherosclerosis in mice^12^,^13^. Given that hepatic FMO3 expression is under the negative control of insulin, hepatic FMO3 gene expression is elevated in obese and insulin resistant mice and, to a lesser extent in obese and patients with diabetes^13^, and fasting glucose levels were found to correlate positively with TMAO levels in humans^2^, the question arises to what extent circulating TMAO levels associate with precisely measured insulin sensitivity in humans. Furthermore, to better understand the impact of TMAO on CVD risk it should be clarified whether circulating TMAO levels associate with cardiovascular risk independently of insulin resistance. Finally, it has not been established whether a lifestyle modification, which is considered an important component in the prevention of CVD^14^,^15^, can reduce increased circulating fasting TMAO levels in humans.

To address these questions we quantified fasting serum TMAO levels in subjects without manifest CVD or kidney disease, which enables to study physiological and pathophysiological relationships under a minimal level of confounding factors. These subjects were at risk for type 2 diabetes, underwent precise measurements of glucose and lipid metabolism and measurement of the carotid intima-media thickness (cIMT) as an early marker of atherosclerosis, and participated in a lifestyle intervention trial.

## Materials and Methods

### Subjects

Caucasians from the southern part of Germany participated in the Tübingen Lifestyle Intervention Program (TULIP)^16^,^17^. Individuals were included into the study when they fulfilled at least one of the following criteria: a family history of type 2 diabetes, a BMI >27 kg/m^2^, previous diagnosis of impaired glucose tolerance or gestational diabetes. Subjects were considered healthy according to a physical examination and routine laboratory tests. As assessed by means of a standard questionnaire, the participants had no history of liver disease such as hepatitis, and did not consume more than >21 drinks (men) and >14 drinks (women) per week, cut-offs that are being considered to represent significant alcohol consumption when evaluating patients with suspected nonalcoholic fatty liver disease (NAFLD)^18^. A total of 220 subjects had precise measurements of body fat distribution, liver fat content and type 2 diabetes biomarkers at baseline and after 9 months of the lifestyle intervention. To investigate determinants of hepatic FMO3 expression and its relationship with insulin resistance we also studied another group of 55 subjects who underwent liver surgery and donated liver samples for research purposes^19^. Informed written consent was obtained from all participants and the Ethics Committee of the University of Tübingen had approved the protocol. The methods were carried out in accordance with the approved guidelines.

### Lifestyle intervention

After the baseline measurements, individuals underwent dietary counselling and had up to ten sessions with a dietician. Subjects presented a three day food intake protocol to each visit. By individual counselling the aim was to reduce the intake of fat to amounts lower than 30% of total calorie intake, to reduce the intake of saturated fat to amounts lower than 10% of total fat intake, to increase the intake of fibres to 15 g/1000 kcal and to achieve a weight loss >5% during the study. Diet composition was estimated with a validated computer program using two representative days of a 3 day diary (DGE-PC 3.0; Deutsche Gesellschaft für Ernährung, Bonn, Germany). Individuals were asked to perform at least 3 hr of moderate sports per week. Aerobic endurance exercise (e.g. walking, swimming) with only moderate increase in the heart rate was encouraged. The anthropometric, metabolic, physical activity and energy intake measurements were repeated after 9 months.

### Habitual physical activity

All individuals completed a standardized self-administered and validated questionnaire to measure physical activity, and a habitual physical activity (HPA) score was calculated^20^.

### Total body fat mass, body fat distribution and liver fat content

Measurements of total body- and visceral fat and mass were performed by an axial T1-weighted fast spin echo technique with a 1.5 T whole-body magnetic resonance imager. Liver fat content was measured by localized proton magnetic resonance (^1^H-MR) spectroscopy as previously described^21^. NAFLD was defined as liver fat content >5.56%^22^.

### Oral glucose tolerance test

Subjects underwent a frequently sampled 2 hr, 75 g oral glucose tolerance test (OGTT). Venous plasma samples were obtained at 0, 30, 60, 90, and 120 minutes for determination of plasma glucose and insulin levels. Whole body insulin sensitivity was calculated from glucose and insulin values during the OGTT as proposed by Matsuda and DeFronzo^23^ or by using the homeostatic model assessment of insulin resistance (HOMA-IR)^24^ in the 55 subjects from whom liver samples were available. Insulin clearance was estimated from the OGTT as C-peptide AUC/insulin AUC.

### Euglycemic, hyperinsulinemic clamp

In a subgroup of 167 subjects insulin sensitivity was also determined during a euglycemic, hyperinsulinemic clamp with a primed insulin infusion at a rate of 40 mU · m^−2^ · min^−1^ for 2 hours. The insulin sensitivity index measured during the clamp (in μmol·kg^−1^ · min^−1^ · pM^−1^) was calculated as the mean infusion rate of glucose (in μmol · kg^−1^ · min^−1^) necessary to maintain euglycemia during the last 40 minutes of the euglycemic hyperinsulinemic clamp divided by the steady state plasma insulin concentration.

### Carotid intima-media thickness

The cIMT was measured in the fasting state using a high-resolution ultrasound system (AU5 idea, Esaote Biomedica, Munich, Germany) with an integrated electrocardiography (ECG) package as previously described^25^.

### Liver samples

A total of 55 patients (20 females/35 males, age 62 ± 11 years, bodyweight 76 ± 13 kg, BMI 25.2 ± 3.9 kg ∙ m^−2^) who underwent liver surgery (e.g. for the resection of solitary hepatic lesions) in the Department of General, Visceral and Transplant Surgery at the University of Tübingen were included in the present study. Patients were fasted overnight prior to collection of the liver samples. Patients tested negative for viral hepatitis and had no liver cirrhosis. Liver samples were taken from normal, non-diseased tissue during surgery, immediately frozen in liquid nitrogen and stored at −80 °C.

### RNA isolation, RT-PCR, and real-time quantitative PCR analysis of hepatic mRNA expression

Frozen tissue was homogenized in a TissueLyser (Qiagen, Hilden, Germany), and RNA was extracted with the RNeasy Tissue Kit (Qiagen, Hilden, Germany) according to the manufacturer’s instructions. Total RNA treated with RNase-free DNase I was transcribed into cDNA using first-strand cDNA kit and PCRs were performed in duplicates on a LightCycler480 (Roche Diagnostics, Mannheim, Germany). Data are presented relative to the housekeeping gene Rps13 using the ΔΔCt method. The human primer sequences used are: for RPS13 up 5′-CCCCACTTGGTTGAAGTTGA-3′ and down 5′-ACACCATGTGAATCTCTCAGGA-3′ and for FMO3 5′-cctgctttgagaagagcaatg-3′ and down 5′- tggaaaagactgatttgtaaatgct-3′.

### Determination of liver tissue triglyceride content

Tissue samples were homogenized in phosphate-buffered-saline containing 1% Triton X-100 with a TissueLyser (Qiagen, Hilden, Germany). To determine the liver fat content, triglyceride and total protein concentration in the homogenate was quantified using the ADVIA 1800 clinical chemistry analyzer (Siemens Healthcare Diagnostics, Eschborn, Germany) and the results were calculated as mg/100 mg tissue (%) or relative to protein content respectively.

### Analytical procedures

Blood glucose was determined using a bedside glucose analyzer (glucose-oxidase method; YSI, Yellow Springs Instruments, Yellow Springs, OH). Plasma insulin was determined on an ADVIA Centaur XP and all other blood parameters on an ADVIA 1800 clinical chemistry system (Siemens Healthcare systems, Erlangen, Germany). The estimated glomerular filtration rate (eGFR) was quantified by using the MDRD-4 equation^26^.

TMAO was measured in serum samples that were stored at −80 °C. Stability studies revealed that TMAO is stable under these storage conditions for several years^27^. For the quantification of serum TMAO levels from samples first sample preparation was performed. The extraction solvent consisted of methanol (MeOH)/acetonitirile (CAN) (v/v, 1/1) containing d9-TMAO (1 μM) and d3-carnitine (20 μM). 200 μL extraction solvent was added to 50 μL serum, vortexed for 1 min and centrifuged at 13,000 g for 10 min. Then 100 μL supernatant was freeze-dried and before analysis redissolved in 1 mL 70% ACN, sonicated for 10 min, then transferred to a sample vial for ultra-high performance liquid chromatography (UHPLC)-MS/MS analysis. The chromatographic separation was performed with a Nexera X2 UHPLC system (Shimadzu, Japan) equipped with a silica hydride column (150 × 2.1 mm, 4 μm, MicroSolv Technology Corporation, USA). The following settings were used: oven temperature 40 °C, flow rate 0.4 mL/min, autosampler temperature 10 °C, injection volume 1 μL. Mobile phase A was 50% ACN containing 0.1% formic acid and 10 mM ammonium formate, mobile phase B was 90% ACN containing 0.1% formic acid. The gradient program started with 50% A, increased to 85% A within 3 min, held 85% A for 2 min, increased to 100% A in 3 min, held this for 2 min, finally decreased back to 50% A in 0.5 min for an equilibration of 2.5 min. Mass spectrometric analysis was performed using a triple quadrupole 8050 electrospray ionization (ESI)-MS (Shimadzu, Japan) in the positive ion mode. The ESI source parameters were: nebulizing gas flow 3 L/min, heating gas flow 10 L/min, interface temperature 300 °C, desolvation line temperature 250 °C, heat block temperature 400 °C, drying gas flow 10 L/min. Multiple Reaction Monitoring (MRM) was used. TMAO and carnitine was quantified by deuterated internal standards.

### Statistical analyses

Data that were not normally distributed (Shapiro-Wilk *W* test; e.g. cIMT, insulin sensitivity, body fat mass, liver fat, content, blood biomarkers) were logarithmically transformed. Differences between baseline and follow-up were tested using the matched pairs *t test*. Univariate associations between parameters were tested using Pearson correlation analyses. To adjust the effects of covariates and identify independent relationships, stepwise and multivariate linear regression analyses were used. The statistical software package JMP 11.0 (SAS Institute Inc, Cary, NC, USA) was used.

## Results

### Subject characteristics

Anthropometrics and metabolic characteristics of the 220 subjects, who were phenotyped at baseline and at follow-up, are shown in the Table 1. A total of 90 males and 130 females with a mean age of 46 years and a mean BMI of 29.5 kg ∙ m^−2^ were studied. Applying ultra-high performance liquid chromatography mass spectrometry, as was done in previous studies^2^,^4^ mean ± SD baseline fasting levels of serum TMAO of 2.83 ± 1.6 μmol/l in our study were very similar in magnitude as was reported in those studies. There was a large variability in serum TMAO levels among the subjects ranging from 0.77 to 11.51 μmol/l.

### Cross-sectional relationships at baseline

At baseline circulating TMAO levels were not different between males (2.82 ± 1.60 μmol/l) and females (2.85 ± 1.64 μmol/l, p = 0.89). They correlated positively with age, BMI, total- and LDL- cholesterol, apolipoprotein B levels and tumor necrosis factor (TNF) alpha levels (N = 144), but not with total body fat mass, HDL-cholesterol or high sensitivity C-reactive protein (hs-CRP) levels (Table 2).

**Figure 1:**
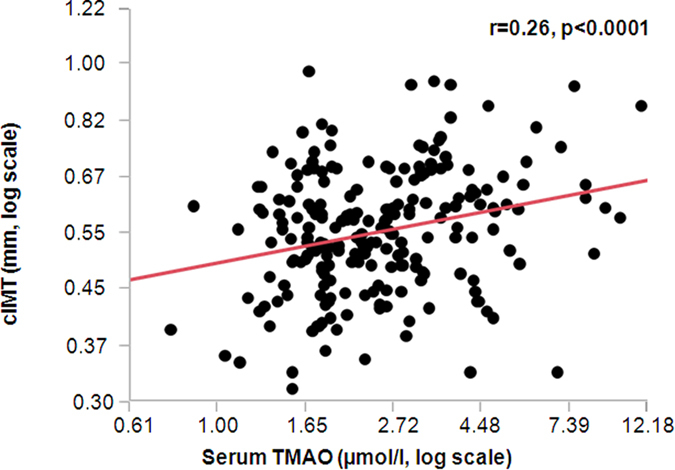
Cross-sectional relationship of fasting serum TMAO levels with cIMT. Univariate cross-sectional relationship (Pearson correlation coefficient and p-value) of fasting serum trimethylamine N-Oxide (TMAO) levels with carotid intima-media thickness (cIMT) in 220 subjects.

Of particular interest were possible relationships of circulating TMAO levels with renal function, body fat distribution, liver fat content, glycemia, insulin sensitivity and cIMT. TMAO levels correlated negatively with the eGFR, tended to associate positively with visceral fat mass and did not associate with liver fat content. While a positive correlation of TMAO levels was found with fasting glycemia, no relationship was observed with 2 hr glucose levels and insulin sensitivity, measured during the OGTT or the euglycemic, hyperinsulinemic clamp (Table 2). There was also no relationship of TMAO levels with insulin clearance. Furthermore, after adjustment for age, sex and BMI no relationship of TMAO levels with insulin sensitivity (OGTT, p = 0.25; clamp, p = 0.69) was found. However, TMAO levels correlated positively with cIMT (Fig. 1).

When we investigated possible relationships of cIMT with anthropometrics and metabolic characteristics we found that a somewhat other picture emerged compared to the relationships of TMAO levels with these parameters. While similar to TMAO levels, cIMT correlated with age, BMI, fasting glucose levels, total- and LDL-cholestreol levels and apolipoprotein B levels, cIMT also correlated with sex (women: 0.54 ± 0.11; men: 0.60 ± 0.13 mm, p = 0.0009), visceral fat mass, liver fat content and insulin sensitivity (Table 2).

We next investigated whether higher TMAO levels associated with increased cIMT independently of established determinants of CVD risk. For this we first ran a forward stepwise linear regression analysis including all parameters that correlated with cIMT. In this analysis age was found to be the strongest determinant of cIMT (p < 0.0001) and was followed by visceral fat mass (p = 0.0001) and TMAO levels (p = 0.03), while all the other parameters were not independent determinants of cIMT. In a multivariate linear regression model including age, visceral fat mass and additionally sex, which strongly associated with cIMT, higher TMAO associated with elevated cIMT, independently of these parameters (Table 3).

### Longitudinal relationships

**Figure 2:**
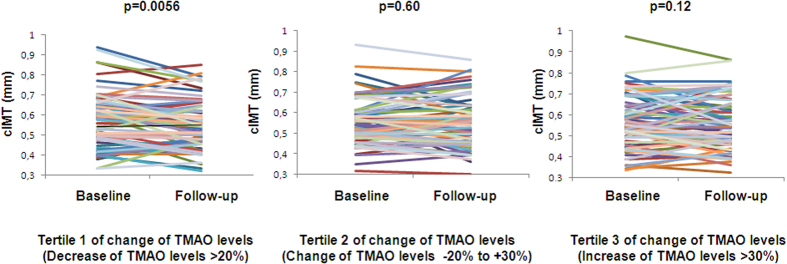
Change of TMAO levels and change of cIMT during 9 months of lifestyle intervention. Individual carotid intima-media thickness (cIMT) before and after 9 months of lifestyle intervention according to tertiles of changes in fasting serum trimethylamine N-Oxide (TMAO) levels (p-value from the paired t test) in 220 subjects.

During the lifestyle intervention most anthropometric and metabolic parameters, which are associated with CVD risk, as well as cIMT improved. Interestingly, mean TMAO levels did not change (Table 1). Mean total energy (from 2014 ± 527 kcal to 1855 ± 393 kcal, p < 0.0001), total fat (from 33 ± 6% to 31 ± 5%, p < 0.0001) and saturated fat (percentage of total fat; from 44 ± 6% to 41 ± 4%, p < 0.0001) intake decreased, yet the changes of these parameters did not correlate with the change of TMAO levels (all p values ≥0.31). Furthermore, no significant relationship was observed between the change of TMAO levels with the change of cIMT in the whole population. However, when we stratified subjects into tertiles of change in TMAO levels (Table 4) we found that cIMT decreased significantly only in subjects in the tertile with the largest decrease of TMAO levels (>20%) (Table 4 and Fig. 2). Interestingly, while in the other two tertiles most parameters considerably improved (e.g. liver fat content, insulin sensitivity) such changes were weaker or absent in the tertile with the largest decrease in TMAO levels (Table 4).

### *FMO3* mRNA expression in liver samples

**Figure 3:**
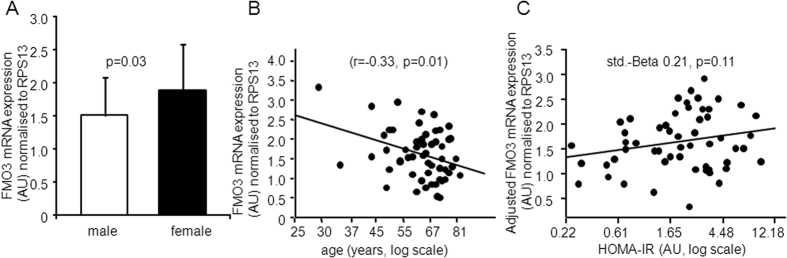
Relationships of hepatic FMO3 mRNA expression with sex, age and adjusted HOMA-IR. Relationship of hepatic FMO3 mRNA expression (normalized for the mRNA expression of the housekeeping gene RSP13) with gender (**A**) and age (**B**) and of hepatic FMO3 mRNA expression, adjusted for sex and gender, with the homeostatic model assessment of insulin resistance (HOMA-IR) in 55 patients who donated liver samples (**C**).

Considering that TMAO levels are not only influenced by the diet but also by FMO3 activity, and that hepatic *FMO3* expression is under the control of insulin signalling, we also investigated whether hepatic *FMO3* mRNA expression associated with anthropometric and metabolic parameters. In 55 liver samples *FMO3* mRNA expression levels were higher in females than in males (Fig. 3, panel A). *FMO3* mRNA expression was not associated with BMI or eGFR (both p > 0.45). However, there was a negative correlation between hepatic *FMO3* mRNA expression and age (Fig. 3, panel B). Liver fat content as well as insulin resistance, measured by HOMA-IR, were not significantly associated with hepatic *FMO3* mRNA expression (both p > 0.26). After adjustment for age and gender, HOMA-IR tended to positively associate with hepatic *FMO3* mRNA expression (Fig. 3, panel C).

## Discussion

Because CVD is the leading cause of deaths in industrialized countries^28^ better prediction and prevention of the disease is of major importance for the individual and the society. In the search for the major pathomechanisms promoting CVD the identification of the meta-organismal pathway involving dietary intake, gut microbiota and liver metabolism has raised much interest in the field of CVD research. Particularly increased generation of TMAO is thought to represent a major pathomechanism contributing to increased CVD risk^1^. While *in vitro* and animal data support that TMAO may play an important role in the pathogenesis of atherosclerosis^29^,^30^, in humans such a role has only been estimated from correlation studies^2^,^3^,^4^,^5^. Recently other studies could not confirm that elevated fasting blood TMAO levels predict increased CVD risk^7^,^8^. It was hypothesized that a positive relationship of TMAO levels with CVD risk may in part be confounded by impaired kidney function and poor metabolic control^8^. Furthermore, because hepatic FMO3, the enzyme that is involved in the generation of TMAO, is under strong control of insulin signalling^13^ and because TMAO is thought to regulate insulin signalling in mice^31^, insulin sensitivity may represent another important confounding factor in such a relationship.

To address these issues we measured fasting serum TMAO levels in our study population that was phenotyped for glucose and lipid metabolism, body fat distribution, ectopic lipid deposition in the liver and cIMT, an early marker of atherosclerosis. In agreement with other studies^2^,^8^ we found TMAO levels to correlate positively with age, BMI, fasting glycemia and blood lipids. Furthermore, although TMAO levels did not associate with hs-CRP levels, there was a relative strong and positive correlation of TMAO levels with TNF-alpha levels, a finding that may warrant further investigation. TMAO levels also correlated positively with cIMT. Interestingly, while insulin sensitivity also associated with cIMT, no relationship was found between TMAO levels and insulin sensitivity, measured by the OGTT or the clamp.

To our knowledge this is the first study investigating possible relationships of TMAO levels with precisely measured insulin sensitivity and early atherosclerosis in a large group of subjects. Our findings support the notion that TMAO levels predict CVD risk independently of insulin sensitivity, a major pathomechanism of cardiometabolic diseases^32^. Furthermore, we provide information about possible effects of insulin sensitivity on endogenous production of TMAO. Studies in insulin receptor knock-out mice or in patients with defects in insulin signalling can help to understand the impact of insulin signalling on gene expression^33^. In this respect recently Miao *et al*. showed that in Liver Insulin Receptor Knockout (LIRKO) mice FMO3 was the second most highly upregulated hepatic transcript and that TMAO was the most strongly upregulated metabolite in these animals, compared to controls^13^. Furthermore, in that study insulin was found to suppress *FMO3* mRNA expression in primary hepatocytes, and it was shown that *FMO3* knockdown improved glucose tolerance in mice. Finally, hepatic *FMO3* mRNA expression was found to be increased in females and in heavily obese and hyperglycemic patients, compared to leaner and normoglycemic subjects^13^. In agreement with Miao *et al*.^13^ in our normal weight patients who donated liver samples we also found hepatic *FMO3* mRNA expression to be elevated in females. Furthermore, we found *FMO3* expression to negatively associate with age and that *FMO3* expression tended to associate positively with adjusted insulin resistance. These data, and the fact that in our larger group of 220 subjects TMAO levels correlated positively with fasting glycemia, an estimate of hepatic insulin resistance, indicates that also in humans fasting TMAO levels appear to be regulated to a considerable extent by hepatic FMO3. However, because TMAO levels did not correlate with whole-body insulin resistance TMAO does not appear to be a major regulator of glucose metabolism in humans.

In agreement with the findings from our *cross-sectional* analysis also in our longitudinal analyses the change of insulin sensitivity did not associate with the change of TMAO levels. Importantly, while insulin sensitivity and other cardiometabolic risk parameters improved, mean TMAO levels did not change. When we investigated whether the change of macronutrient intake and the intake of saturated fat, which is mainly derived from animal sources, correlates with the change of TMAO levels, no such relationship was found. Previous studies investigating the impact of diet modification on fasting TMAO levels revealed that a 2-week high-fat diet did not change fasting TMAO levels^34^, while a small increase of TMAO levels was found during a 4-week high-fat diet intervention^35^. Together with our data these findings indicate that fasting TMAO levels are not strongly regulated by a moderate modification of the diet that is currently being recommended for the prevention of cardiometabolic diseases^36^. However, we have no solid information about the dietary intake of choline or carnitine in our study and, therefore, cannot comment whether a specific modification of foods rich in choline or carnitine occurred in some of our subjects. Furthermore, we have no information about the gut microbial composition and function of our subjects. It is important to study these aspects in future studies, because Hazen’s group very recently elegantly showed in mice that the microbial TMA lyase activity is very important for the conversion of choline and carnitine into TMA^37^. Individual differences in the gut microbiome and the nutrient intake may have resulted in that we found no significant mean change of fasting TMAO levels during our intervention, yet a large variability in the change of TMAO levels, ranging from a decrease of 82% to an increase of 730%.

When we stratified our subjects into tertiles of the change of TMAO levels we found that cIMT decreased significantly only in subjects in the tertile with the largest decrease of TMAO levels. Interestingly, specifically in this tertile, except for TMAO levels, the improvement of many metabolic parameters (e.g. insulin sensitivity and glycemia), liver fat content and viscveral fat mass was smallest, indicating that the improvement of cIMT in this tertile was most probably not strongly affected by the improvement of these parameters. Otherwise, one may expect cIMT not to decrease, or even increase, in the tertile where TMAO levels increased. However, particularly in this tertile the improvement of insulin sensitivity, glycemia, liver fat content visceral fat mass and serum lipids was largest, which may have counteracted the effect of increasing TMAO levels on cIMT. Of note, most studies investigating the change of cIMT during lifestyle or pharmacological interventions, or during a natural follow-up, found that, based on low reversibility of arterial structure, cIMT did not decrease, but that the intervention or a healthy lifestyle resulted in a reduced progression of cIMT^38^,^39^. The fact that mean cIMT decreased during the intervention in our study indicates that lifestyle modification is effective to revert atherosclerosis, particularly in early stages of its pathophysiological process.

In summary, with this report we provide novel information, particularly from a metabolic angle, that may help to better understand the role of TMAO in the natural history of cardiometabolic diseases. Based on the relationships of fasting TMAO levels with cIMT in the *cross-sectional* and longitudinal analyses, our data support the conclusion that derived from published animal and human data, that TMAO levels have an important role in the pathophysiology of CVD. Furthermore, we found that fasting TMAO levels associated positively with fasting glycemia and that hepatic *FMO3* mRNA expression tended to associate positively with the HOMA-IR. These findings indicate that, similar as shown in animals and in obese, hyperglycemic humans^13^ *FMO3* expression and TMAO levels appear to be increased in hepatic insulin resistance. However, and in contrast to other liver-derived proteins, such as the hepatokine fetuin-A^40^, TMAO does not appear to be a major regulator of whole-body insulin sensitivity. Finally, the fact that mean TMAO levels did not change during a standard lifestyle intervention, which is effective to improve most of the studied cardiometabolic risk parameteres, indicates that it is important to better understand mechanisms regulating TMAO blood levels.

In conclusion, we found that increased fasting serum TMAO levels associate with increased cIMT, independently of established cardiovascular risk markers, including insulin resistance, visceral obesity and fatty liver. Furthermore, the decrease of cIMT during a lifestyle intervention appears to be related to the decrease of TMAO levels. Our data support the idea that further research into this meta-organismal pathway may help to better understand the natural history of CVD.

## Additional Information

**How to cite this article**: Randrianarisoa, E. *et al*. Relationship of Serum Trimethylamine N-Oxide (TMAO) Levels with early Atherosclerosis in Humans. *Sci. Rep.* **6**, 26745; doi: 10.1038/srep26745 (2016).

## Figures and Tables

**Table 1:** Anthropometric and metabolic parameters at baseline and at follow-up.

	Baseline	Follow-up	p-value
Gender (males/females)	90/130		
Age (years)	46 ± 11		
Body mass index (kg ∙ m^−2^)	29.5 ± 4.8	28.6 ± 4.7	<0.0001
Total body fat_MRT_ (kg)	26.2 ± 10.3	23.7 ± 10.1	<0.0001
Visceral fat_MRT_ (kg)	3.1 ± 1.9	2.6 ± 1.8	<0.0001
Liver fat_MRS_ (%)	5.8 ± 6.6	4.2 ± 5.4	<0.0001
Fasting glucose (mM)	5.3 ± 0.5	5.2 ± 0.5	0.01
Fasting insulin (pM)	63 ± 44	55 ± 34	<0.0001
2 hr glucose (mM)	7.0 ± 1.5	6.7 ± 1.7	0.007
Total-cholesterol (mg/dl)	194 ± 36	192 ± 37	0.05
HDL-cholesterol (mg/dl)	53 ± 13	53 ± 14	0.20
LDL-cholesterol (mg/dl)	121 ± 30	118 ± 33	0.02
Triglycerides (mg/dl)	126 ± 110	118 ± 96	0.03
Apolipoprotein B (mg/dl)	95 ± 22	92 ± 21	0.006
Hs-CRP (mg/dl)	0.22 ± 0.31	0.17 ± 0.25	<0.0001
TNF-alpha (pg/ml)*	3.0 ± 7.6	1.3 ± 2.8	0.0004
Systolic BP (mmHg)	124 ± 16	122 ± 15	0.04
Diastolic BP (mmHg)	77 ± 11	75 ± 10	0.02
eGFR (ml/min/1.73 m^2^)	80.1 ± 16.1	83.5 ± 17.9	<0.0001
HOMA-IR (arb. u.)	2.5 ± 1.9	2.1 ± 1.4	<0.0001
Insulin sensitivity_OGTT_ (arb. u.)	12.7 ± 7.1	14.3 ± 7.7	<0.0001
Insulin sensitivity_Clamp_ (μmol·kg^−1^·min^−1^·pM^−1^)#	0.066 ± 0.039	0.079 ± 0.050	0.06
Insulin clearance (arb. u.)	5.52 ± 1.91	5.72 ± 1.88	0.007
cIMT (mm)	0.57 ± 0.12	0.55 ± 0.12	0.02
TMAO (μmol/l)	2.83 ± 1.62	3.2 ± 2.89	0.18

Data are means ± SD.

^*^Only available in 144 subjects.

^#^Only available in 167 subjects at baseline and in 45 subjects at follow-up. MRT: magnetic resonance tomography, MRS: magnetic resonance spectroscopy; eGFR: estimated glomerular filtration rate; HOMA-IR: homeostatic model assessment of insulin resistance; cIMT: carotid intima-media thickness; TMAO: trimethylamine N-oxide; BP: blood pressure.

**Table 2:** Relationships of cIMT and fasting TMAO levels with selected parameters at baseline.

	TMAO levels		cIMT	
	r	p	r	p
Age	**0.23**	**0.0006**	**0.64**	**<0.0001**
Body mass index	**0.14**	**0.03**	**0.19**	**0.004**
Total body fat _MRT_	0.07	0.27	0.06	0.42
Visceral fat_MRT_	0.12	0.07	**0.40**	**<0.0001**
Liver fat_MRS_	0.05	0.45	**0.23**	**0.0005**
Fasting glucose	**0.15**	**0.02**	**0.25**	**0.0002**
Fasting insulin	0.02	0.72	0.12	0.07
2 hr glucose	−0.08	0.25	**0.18**	**0.008**
Total cholesterol	**0.14**	**0.04**	**0.17**	**0.01**
HDL-cholesterol	0.01	0.84	−0.12	0.08
LDL-cholesterol	**0.14**	**0.03**	**0.12**	**0.005**
Triglycerides	0.07	0.29	0.12	0.07
Apolipoprotein B	**0.18**	**0.009**	**0.27**	**<0.0001**
Hs-CRP	−0.01	0.88	0.13	0.06
TNF-alpha*	**0.27**	**0.001**	0.10	0.22
Systolic BP	0.006	0.93	**0.19**	**0.005**
Diastolic BP	−0.005	0.94	0.12	0.09
eGFR	**−0.14**	**0.04**	−0.06	0.36
HOMA-IR	0.05	0.48	**0.15**	**0.02**
Insulin sensitivity_OGTT_	−0.002	0.98	**−0.18**	**0.009**
Insulin sensitivity_Clamp_#	−0.03	0.75	−0.13	0.10
Insulin clearance	0.03	0.62	−0.11	0.12

r, Pearson correlation coefficient; *Only available in 144 subjects. ^#^Only available in 167 subjects. BIA, body impedance; MRT, magnetic resonance tomography; MRS, magnetic resonance spectroscopy. MRT: magnetic resonance tomography, MRS: magnetic resonance spectroscopy; eGFR: estimated glomerular filtration rate; HOMA-IR: homeostatic model assessment of insulin resistance; cIMT: carotid intima-media thickness; TMAO: trimethylamine N-oxide; BP: blood pressure.

**Table 3:** Determinants of cIMT in multivariate linear regression models.

Covariates	Estimate ± SE	t-value	std.−Beta	p
Female sex	−0.02 ± 0.01	−1.14	−0.07	0.25
Age	0.45 ± 0.04	10.31	0.56	<0.0001
Visceral fat mass	0.05 ± 0.02	2.45	0.16	0.02
TMAO levels	0.05 ± 0.02	2.15	0.11	0.03

**Table 4:** Anthropometric and metabolic parameters in tertiles of changes of TMAO levels during the lifestyle intervention.

	1^st^ Tertile		2^nd^ Tertile		3^rd^ Tertile	
	Baseline	Follow-up	Baseline	Follow-up	Baseline	Follow-up
TMAO (μmol/l)	3.84 ± 2.06	2.1 ± 0.80***	2.54 ± 1.11	2.57 ± 1.07	2.13 ± 0.94	4.82 ± 4.43***
cIMT (mm)	0.58 ± 0.13	0.55 ± 0.12*	0.56 ± 0.11	0.56 ± 0.12	0.56 ± 0.13	0.55 ± 0.12
Gender (males/females)	26/47		29/46		35/37	
Age (years)	44 ± 12		47 ± 11		48 ± 10	
Body mass index (kg ∙ m^−2^)	29.8 ± 5.2	29.2 ± 5.0**	29.5 ± 4.6	28.5 ± 4.7***	29.3 ± 4.8	27.9 ± 4.4***
Total body fat _MRT_ (kg)	26.7 ± 11.3	25.1 ± 10.9**	26.7 ± 9.8	24.1 ± 9.7***	25.1 ± 9.7	21.8 ± 9.6***
Visceral fat _MRT_ (kg)	2.8 ± 1.8	2.5 ± 1.7***	3.0 ± 1.8	2.6 ± 1.8***	3.3 ± 2.0	2.7 ± 1.***
Liver fat _MRS_ (%)	5.6 ± 6.3	4.5 ± 6.6*	4.9 ± 5.4	3.9 ± 4.9**	6.9 ± 7.8	4.2 ± 4.4***
Fasting glucose (mM)	5.2 ± 0.5	5.2 ± 0.5	5.2 ± 0.5	5.2 ± 0.6	5.3 ± 0.6	5.2 ± 0.5*
**Fasting insulin (pM)**	**64 **±** 42**	**62 **±** 38**	**60 **±** 34**	52 ± 32**	**66 **±** 54**	**50 **± 32**
2 hr glucose (mM)	6.7 ± 1.4	6.5 ± 1.6	6.9 ± 1.5	6.9 ± 1.8	7.3 ± 1.7	6.8 ± 1.6**
Total cholesterol (mg/dl)	193 ± 36	193 ± 36	188 ± 37	185 ± 38	201 ± 37	198 ± 38
HDL-cholesterol (mg/dl)	54 ± 14	54 ± 12	50 ± 11	50 ± 12	54 ± 14	55 ± 16
LDL-cholesterol (mg/dl)	120 ± 31	121 ± 30	117 ± 29	113 ± 37	125 ± 29	119 ± 32*
Triglycerides (mg/dl)	117 ± 101	110 ± 62	117 ± 72	110 ± 71	145 ± 144	134 ± 138
Apolipoprotein B (mg/dl)	94 ± 21	91 ± 18	92 ± 22	91 ± 23	99 ± 23	93 ± 22**
Hs-CRP (mg/dl)	0.26 ± 0.38	0.18 ± 0.27**	0.20 ± 0.26	0.19 ± 0.28	0.21 ± 0.28	0.15 ± 0.21*
TNF-alpha (pg/ml)^§^	2.9 ± 6.7	1.8 ± 4.4*	4.0 ± 10.0	1.3 ± 2.2*	2.2 ± 5.5	0.8 ± 0.4*
Systolic BP (mmHg)	121 ± 16	120 ± 16	127 ± 17	124 ± 16	124 ± 15	123 ± 14
Diastolic BP (mmHg)	76 ± 11	75 ± 10	77 ± 11	76 ± 10	77 ± 12	76 ± 10
eGFR (ml/min/1.73m^2^)	82.1 ± 17.7	85.7 ± 19.3*	78.5 ± 16.1	83.8 ± 18.2**	79.7± 14.3	82.1 ± 16.1
HOMA-IR (arb. u.)	2.5 ± 1.7	2.4 ± 1.5	2.4 ± 1.4	2.0 ± 1.3**	2.7 ± 2.5	1.9 ± 1.4**
Insulin sensitivity_OGTT_ (arb. u.)	12.7 ± 6.8	12.9 ± 6.8	12.8 ± 7.0	14.8 ± 7.8**	12.6 ± 7.5	15.3 ± 8.3***
**Insulin clearance (arb. u.)**	**5.41 **±** 1.85**	**5.36 **±** 1.88**	**5.47 **±** 1.69**	**5.72 **±** 1.70**	**5.66 **±** 2.19**	**6.09 **± 2.00**

Data are means ± SD. ^§^Only available in 144 subjects. ^#^Only available in 167 subjects at baseline and in 45 subjects at follow-up. MRT: magnetic resonance tomography, MRS: magnetic resonance spectroscopy; eGFR: estimated glomerular filtration rate; HOMA-IR: homeostatic model assessment of insulin resistance; cIMT: carotid intima-media thickness; TMAO: trimethylamine N-oxide; BP: blood pressure; *p < 0.05; **p < 0.01; ***p < 0.0001.
